# Continuous renal replacement therapy attenuates endothelial injury biomarkers in pediatric sepsis: a prospective cohort study

**DOI:** 10.1080/0886022X.2026.2639791

**Published:** 2026-03-31

**Authors:** Huijie Miao, Chunxia Wang, Yun Cui, Xi Xiong, Yuqian Ren, Zhibao Lv, Yucai Zhang

**Affiliations:** a Department of Critical Care Medicine, Shanghai Children’s Hospital, Shanghai Jiao Tong University School of Medicine, Shanghai, China; b Institute of Pediatric Critical Care, Shanghai Jiao Tong University, Shanghai, China; c Institute of Pediatric Infection, Immunity, and Critical Care Medicine, Shanghai Children’s Hospital, Shanghai Jiao Tong University School of Medicine, Shanghai, China; d Department of General Surgery, Shanghai Children’s Hospital, Shanghai Jiao Tong University School of Medicine, Shanghai, China

**Keywords:** Pediatric sepsis, continuous renal replacement therapy (CRRT), endothelial biomarkers, vascular permeability, vimentin, critical care nephrology

## Abstract

Endothelial cell (EC) injury is a critical factor in sepsis-induced organ failure. Continuous renal replacement therapy (CRRT) is used to improve hemodynamically unstable sepsis. We conducted a single-center, prospective, observational cohort study to assess whether CRRT attenuates sepsis-associated with EC injury. Based on whether CRRT was implemented within 24 h after admission, patients were divided into non-CRRT and CRRT groups. Demographic data, clinical features, and laboratory indexes were collected, and blood samples were collected at admission, 24 h and 7 days after PICU admission. The levels of vascular endothelial growth factor [VEGF], serum intercellular adhesion molecule-1 [sICAM-1], serum vascular cell adhesion molecule-1 [sVCAM-1], vimentin [VIM]), tissue plasminogen activator-plasminogen activator inhibitor-1 complex [t-PAIC], and thrombomodulin [TM] were not different between the CRRT and non-CRRT groups at PICU admission. The mixed-effects models revealed a significant decreasing trend in EC injury biomarkers (sICAM-1, sVCAM-1, VEGF, and VIM). Nevertheless, there were no obvious changes in these indicators in the non-CRRT group. In the CRRT group, EC injury indicators correlated with albumin and lactic acid at sepsis diagnosis. Serum VEGF, VIM, TM, and t-PAIC levels, but not sVCAM-1 or sICAM-1, were significantly lower in survivors than in non-survivors. Patients with higher serum VEGF, VIM, TM, and t-PAIC levels were at risk of worsening pediatric sepsis outcomes. CRRT downregulated the serum levels of EC injury indicators. This is the first prospective pediatric study linking CRRT to endothelial recovery, and EC biomarkers may serve as surrogate markers for endothelial recovery during CRRT.

## GRAPHICAL ABSTRACT

**Figure:**
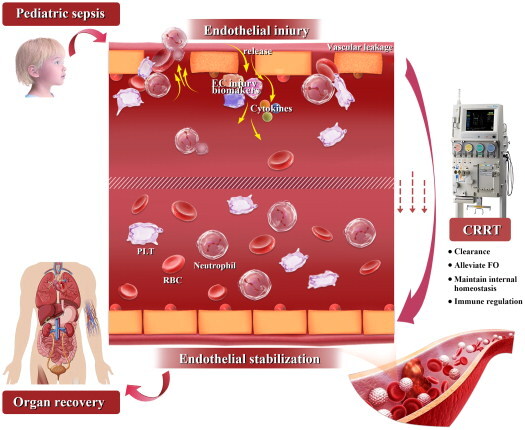

## Introduction

Sepsis is a life-threatening organ dysfunction that arises from dysregulated host response to infection. The reported mortality rates of pediatric sepsis remain high, reaching 23.0% for sepsis and increasing to 36.8% for septic shock in JAMA and CCM [1,2]. The inflammatory response triggers endothelial cell lining (ECL) dysfunction, resulting in vascular leakage, tissue edema, or dysregulation of hemostasis, further leading to life-threatening organ failure during sepsis [3]. Endothelial cell (EC) injury is the key risk factor in sepsis-induced organ failure [3,4]. Therefore, appropriate interventions to attenuate EC dysfunction may reduce mortality in patients with sepsis.

Increased vascular permeability at sites of EC dysfunction is the primary cause of shock and fluid overload (FO) in sepsis [5]. Accumulated evidence indicates that FO and shock were related to high mortality in patients with sepsis [6,7]. The Surviving Sepsis Campaign (SSC) suggested that continuous renal replacement therapy (CRRT) promotes the management of fluid balance in sepsis-induced acute kidney injury patients with hemodynamic instability [1,8]. Therefore, the achievement of hemodynamic stability and fluid balance by CRRT may be associated with the repair of EC dysfunction, contributing to recovery from sepsis. Our previous research indicated that CRRT improves organ dysfunction and outcomes in pediatric sepsis by reducing polymorphonuclear myeloid-derived suppressor cell (PMN-MDSC) expansion or by decreasing serum levels of tumor necrosis factor-α (TNF-α) and interleukin-6 (IL-6) [9–12]. However, the relationship between CRRT and EC function in pediatric sepsis remains unclear.

Given the critical role of hemodynamic instability in the occurrence of EC injury, we aimed to assess the effect of CRRT on sepsis-associated EC injury by monitoring changes in biomarkers of endothelial injury in this pilot prospective observational study.

## Materials and methods

### Study design and patients

We performed a single-center, prospective observational cohort study in pediatric sepsis, and all of these patients who met the criteria for indications for CRRT support and were admitted to the pediatric intensive care unit (PICU) were eligible and enrolled. Ethical approval was obtained from the Institutional Review Board (Ethics Committee of Shanghai Children’s Hospital, Approval number: 2019R032-F01), and the study was conducted in accordance with the Declaration of Helsinki 2013. Written informed consent was obtained from the participants and their legal guardians before participating in the study.

Patients with pediatric sepsis were enrolled between December 2020 and December 2023. Pediatric sepsis was defined based on the criteria established at the International Pediatric Sepsis Consensus Conference in 2005 [13]. The inclusion criteria were as follows: (1) diagnosis of pediatric sepsis within 24 h, (2) age between 28 days and 18 years, and (3) meeting the standards of indications for CRRT. The indications for initiation of CRRT included hyperinflammatory response, hemodynamic instability, fluid overload (FO) >10% (positive FO = weight gain from admission), excessively high ammonia levels (>100 μmol/L), or acute kidney injury (AKI) at stage 2 of KDIGO classification (more than 2-fold increase of sCr level compared with the baseline value and/or urine output <0.5 mL/kg/h for 12 h). AKI was defined as an infectious episode of AKI according to the KDIGO Improving Global Outcomes criteria [14]. Percentage of FO = (∑ daily (fluid intake (L) − total output (L))/body weight (in kilograms)) × 100% [15]. Hemodynamic instability is defined as [13]: despite administration of isotonic intravenous fluid bolus ≥40 mL/kg in 1 h.

- Decreased blood pressure (hypotension) <5th percentile for age or systolic blood pressure <2*SD* below normal for age OR
- Vasoactive drugs need to maintain blood pressure within the normal range (dopamine >5 μg/kg/min or dobutamine, epinephrine, or norepinephrine at any dose) OR
- Increased arterial lactate level >2 mmol/L OR
- Oliguria: urine output <0.5 mL/kg/h OR
- Prolonged capillary refill: >5 s OR
- Core to peripheral temperature gap >3 °C.

Exclusion criteria were lack of consent, PICU stay of <24 h, advanced tumor, congenital heart disease, autoimmune disorders, primary immune function disorder, micronutrient deficiencies, and malnutrition. The patients did not receive CRRT if they met the following criteria: (1) without consent for CRRT and (2) with difficulty in carrying out CRRT included biofilm allergy and extremely serious coagulation dysfunction. Patients were classified into the CRRT and non-CRRT groups according to whether they received CRRT. All patients received standardized intensive care treatment as recommended by the International Guidelines of SSC [16]. The patients in the non-CRRT group were treated with conventional therapy, including antibiotics, fluid substitution, vasopressor treatment, mechanical ventilation, and other care bundles. Organ supportive treatment was provided when necessary, excluding intermittent dialysis. The interval between PICU admission and CRRT initiation was set within 24 h to avoid bias in this study. By default, the blood flow rate for CRRT was maintained at 3–6 mL/kg/min, with a delivered dose of 35 mL/kg/h. For the patient complicated with AKI, hemofiltration and hemodialysis were performed. The dialysate dose was set within a range of 0–20 mL/kg/h. The rates of replacement fluid, dialysate, and ultrafiltration were adjusted according to the patients’ diagnoses, fluid overload, and hemodynamic parameters. An AEF-03 or AEF-07 polysulfone membrane hemofilter (AsahiKASEI) was used, depending on body weight.

The CRRT group was further divided into survivor and non-survivor groups based on their clinical outcome at discharge.

### Data collection and blood sample collection

Data included patient demographics, illness severity, comorbidities, laboratory data, and outcomes. Disease severity was assessed using the Pediatric Risk of Mortality (PRISM) score III and Pediatric Sequential Organ Failure Assessment score (pSOFA). Laboratory parameters included white blood cells (WBC), C-reactive protein (CRP), procalcitonin (PCT), platelets (PLT), lactic acid (Lac), albumin (ALB), serum creatinine (sCr), lactate dehydrogenase (LDH), prothrombin time (PT), activated partial thromboplastin time (APTT), tissue plasminogen activator inhibitor-1 complex (t-PAIC), and thrombomodulin (TM). The other laboratory indices included immune-related indicators: natural killer [NK] cells, B cells (cluster of differentiation [CD]19^+^), percentage of peripheral blood T-cell subsets (CD4^+^CD8^+^), and cytokines (IL-1β, IL-6, IL-8, IL-10, and TNF-α).

Serum samples were collected and stored at −80 °C. Indicators of EC injury (serum intercellular adhesion molecule-1, sICAM-1; serum vascular cell adhesion molecule-1, sVCAM-1; Vimentin, VIM; vascular endothelial growth factor, VEGF) were determined using enzyme-linked immunosorbent assay (ELISA, Human sICAM-1/CD54 ELISA Kit, Catalog Number. 70-EK189-96; Human sVCAM-1/CD106 ELISA Kit, Catalog Number. 70-EK190-96: Human VEGF ELISA Kit, Catalog Number. 70-EK183-96; Human Vimentin ELISA Kit, Catalog Number. ZC-CH004-96T, MultiSciences [LIANKE] Biotech, Co., LTD, Hangzhou, China), according to the manufacturer’s instructions. The samples for the experimental conditions were run in duplicate.

Serum indicators of EC injury, including sICAM-1, sVCAM-1, VEGF, VIM, TM, and t-PAIC were collected before (pre-CRRT) and after CRRT intervention at 24 h (CRRT 24 h) and 7 days (CRRT 7 days) in the CRRT groups. In the non-CRRT groups, serum indicators of EC injury were collected at admission to the PICU and confirmation of the presence of sepsis, 24 h after diagnosis of sepsis, and 7 days after diagnosis of sepsis. The primary outcome of this study was the change in EC injury biomarkers on day 7 (CRRT 7 days or 7 days after diagnosis of sepsis). The secondary outcome was the in-hospital mortality.

### Statistical analysis

The means and standard deviations of normally distributed continuous variables are shown (SDs). Nonparametric variables are presented as medians with interquartile ranges (*IQR*). Categorical data are expressed as frequencies. Significant differences for continuous variables were assessed using Student’s *t*-test or Mann-Whitney *U* test. Differences in the qualitative variable data were compared using the *Chi*-square test or Fisher’s exact test. A multivariate binary logistic regression model was used to adjust for baseline PRISM III score, pSOFA score, shock presence, and AKI to mitigate confounding between CRRT *versus* non-CRRT groups. A linear mixed-effects model was fitted to assess the impact of CRRT on EC injury biomarkers levels over time. The model included the fixed effect of time (pre-CRRT, 24 h post-CRRT, and 7 days post-CRRT) and a random intercept for each patient to account for within-subject correlation due to repeated measurements. Post-hoc pairwise comparisons between time points were performed using the Bonferroni method to adjust for multiple testing. To compare the values between two specific paired time points, the paired t-test was used for normally distributed data, and the Wilcoxon test was used for non-normally distributed data. To further assess the trend of EC injury indicators, we employed both percentage reduction and Cohen’s d (Supplementary Table 2). Sensitivity analyses were conducted to test the robustness of the findings regarding EC injury biomarkers (Supplementary Tables 3–14). Correlation analysis was performed using linear regression. All data were analyzed using STATA and GraphPad Prism software. *p* Was set at *p* < 0.05.

**Table 3.** Changes of biochemical indexes during CRRT in pediatric patients with sepsis.

	Pre-CRRT (*N* = 26)	CRRT after 24 h (*N* = 26)	CRRT after 7 days (*N* = 20)	*p-*Value
WBC (×10^9^/L)	7.52 (3.86, 14.61)	7.96 (4.77, 14.95)	9.99 (6.49, 13.96)	0.574
PLT (/L)	119.50 × 10^9^ (46.00 × 10^9^, 286.00 × 10^9^)	64.50 × 10^9^ (23.00 × 10^9^, 234.00 × 10^9^)	259.00 × 10^9^ (94.00 × 10^9^, 341.00 × 10^9^)^#^	0.080
CRP (mg/L)	100.00 (63.00, 170.00)	82.00 (41.00, 159.00)	16.50 (6.00, 30.50)*^#^	<0.001
PCT (ng/ml)	6.59 (0.41, 8.60)	2.30 (0.84, 6.21)	0.25 (0.15, 0.91)*^#^	0.002
ALB (g/L)	33.90 ± 5.67	39.71 ± 5.88*	41.94 ± 5.22*	<0.001
sCr (μmol/L)	31.50 (25.00, 45.00)	27.00 (21.00, 43.00)	23.00 (17.50, 29.50)*^#^	0.008
LDH (IU/L)	386.50 (235.00, 643.00)	498.00 (315.00, 987.00)	449.00 (314.00, 762.00)	0.334
Lac (mmol/L)	2.00 (1.40, 3.60)	1.75 (1.10, 4.00)	0.85 (0.60, 1.55)*^#^	0.001
PT (s)	14.95 (12.90, 18.70)	15.25 (13.10, 17.43)	12.90 (12.30, 14.10)	0.109
APTT (s)	51.15 (37.40, 68.20)	76.10 (37.50, 138.10)*	27.3 (25.90, 34.60)*^#^	<0.001

WBC: white blood cell; PLT: platelet; CRP: C-reactive protein; PCT: procalcitonin; ALB: albumin; sCr: serum creatinine; LDH: lactate dehydrogenase; Lac: lactate; PT: prothrombin time; APTT: activated partial thromboplastin time.

Values are expressed as the mean ± standard deviation or median (IQR).

*p < 0.05 indicates the significant difference compared with Pre-CRRT group.

^#^*p* < 0.05 indicates the significant difference compared with CRRT after 24 h group.

## Results

**Figure 1.**
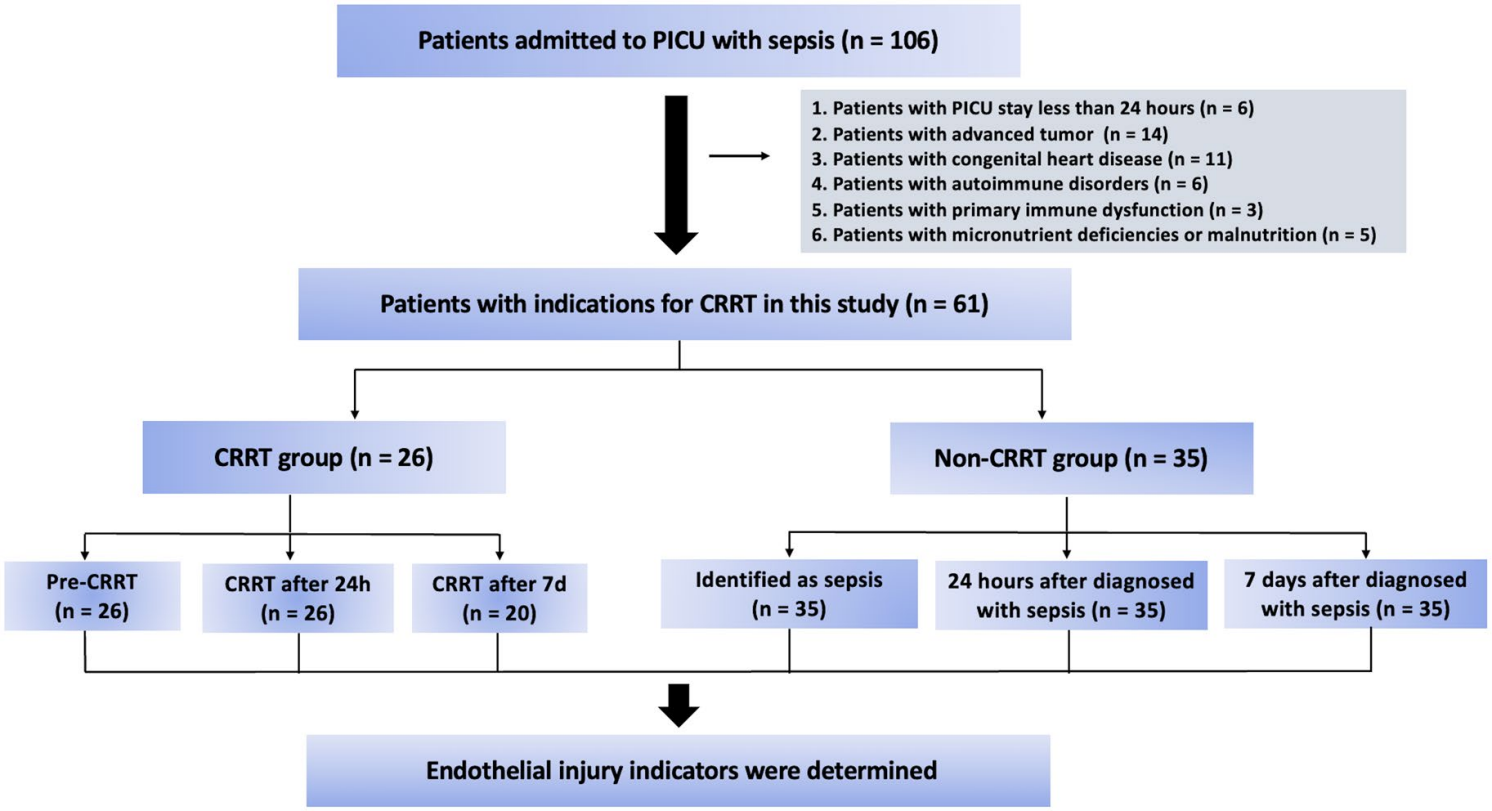
Flowchart of patients’ enrollment in this study.

A total of 106 children with sepsis were enrolled in this study. We excluded 6 patients who died within 24 h after PICU admission, 14 patients who had advanced tumors, 11 patients who had congenital heart disease, 6 patients who had autoimmune disorders, 3 patients who had primary immune dysfunction, and 5 patients who had micronutrient deficiencies or malnutrition. Thus, a total of 61 participants were included in the analysis. Among them, 26 patients (42.62%) received CRRT (CRRT group), and 35 (57.38%) received conventional management (non-CRRT group). There were 6 non-survivors and 20 survivors in the CRRT group (Figure 1). The basic demographic and clinical parameters of the 61 patients are summarized in Table 1.

**Table 1.** Demographic and clinical characteristics of pediatric sepsis at PICU admission.

Characteristics	Total (*N* = 61)	CRRT (*N* = 26)	Non-CRRT (*N* = 35)	95%*CI*	*p-*Value
Age, month	26 (7, 60)	49 (16, 61)	12 (4, 48)	10.56 to 63.44	0.064
Male, *n* (%)	32 (52.46)	15 (57.69)	17 (48.57)	0.46 to 4.55	0.481
PRISM III	14.67 ± 6.43	16.08 ± 6.83	13.63 ± 6.00	−5.75 to 0.85	0.143
pSOFA	10.10 ± 4.98	11.31 ± 5.69	9.20 ± 4.24	−4.65 to 0.43	0.103
**Co-morbidities**					
Hereditary metabolic disease, *n* (%)	11 (18.03)	6 (23.08)	5 (14.29)	0.39 to 8.48	0.377
Trauma, *n* (%)	3 (4.92)	2 (7.69)	1 (2.86)	0.14 to 171.96	0.388
**Organ dysfunction**					
Shock, *n* (%)	25 (40.98)	11 (42.31)	14 (40.00)	0.35 to 3.47	0.856
ARDS, *n* (%)	27 (44.26)	11 (42.31)	16 (45.71)	0.28 to 2.73	0.791
AKI, *n* (%)	14 (22.95)	6 (23.08)	8 (22.86)	0.25 to 3.95	0.984
ALI, *n* (%)	13 (21.31)	8 (30.77)	5 (14.29)	0.64 to 11.88	0.120
Brain dysfunction, *n* (%)	21 (34.43)	10 (38.46)	11 (31.43)	0.41 to 4.50	0.568
Gastrointestinal disorder, *n* (%)	25 (40.98)	10 (38.46)	15 (42.86)	0.26 to 2.64	0.730
Length of PICU stay, days	11 (6, 16)	14 (9, 23)	8 (6, 13)	0.46 to 11.54	0.007
Length of Hospital stay, days	20 (13, 28)	25.5 (15, 37)	17 (13, 25)	0.46 to 16.54	0.053
Hospital mortality, *n* (%)	17 (27.87)	6 (23.08)	11 (31.42)	−1.38 to 0.832	0.472

ARDS: acute respiratory distress syndrome; AKI: acute kidney injury; ALI: acute liver injury; PICU: pediatric intensive care unit; PRISM III: pediatric risk of mortality score III; pSOFA: pediatric sequential organ failure assessment score.

Values are expressed as the median ± standard deviation or median (IQR) or *n* (%).

### Characteristics of pediatric sepsis enrolled at the PICU

Thirty-two males and 29 females with a median age of 26 (7, 60) months were included in this prospective study, and the overall hospital mortality rate was 27.87% (17/61). After adjusting for the aforementioned confounders (baseline PRISM III score, pSOFA score, shock presence, and AKI), the association between CRRT and the outcome was no longer statistically significant (adjusted OR = 2.59, 95% *CI*: 0.60–11.27, *p* = 0.204). There was no significant difference in hospital mortality rates between the CRRT group (23.08% [6/26]) and the non-CRRT group (31.42% [11/35]) were not significantly different (*p* = 0.472) (Table 1). In addition, the length of PICU stay was 14 (9, 23) days in the CRRT group and 8 (6, 13) days in the non-CRRT group, with a significant difference (*p* = 0.007) (Table 1). However, there were no obvious changes in the incidence of shock between the CRRT and non-CRRT groups (*p* = 0.856), similar to AKI (*p* = 0.984), acute respiratory distress syndrome (ARDS) (*p* = 0.791), acute liver injury (ALI) (*p* = 0.112), brain dysfunction (*p* = 0.568), or gastrointestinal disorder (*p* = 0.730) (Table 1).

### Variables of endothelial injury in CRRT and non-CRRT groups

**Figure 2.**
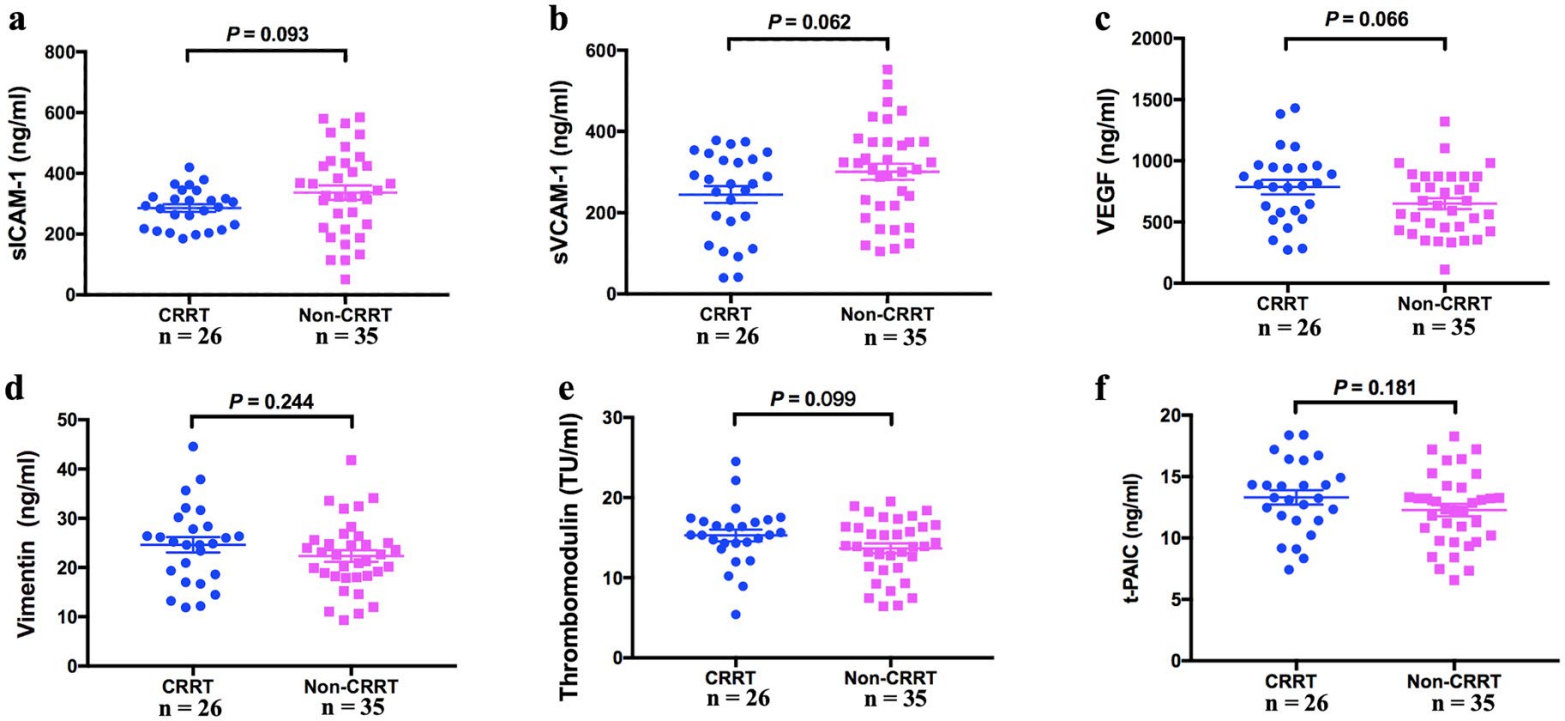
Serum indicators of endothelial cell injury between CRRT and Non-CRRT groups at sepsis diagnosis. (a) sICAM-1; (b) sVCAM-1; (c) VEGF; (d) Vimentin; (e) TM; (f) t-PAIC.

At PICU admission and identified as sepsis within 24 h, there were no obvious changes in the levels of sICAM-1, sVCAM-1, VEGF, VIM, TM, and t-PAIC between the non-CRRT and CRRT groups (*p* = 0.093, *p* = 0.062, *p* = 0.066, *p* = 0.244, *p* = 0.099, and *p* = 0.181, respectively) (Figure 2).

**Figure 3.**
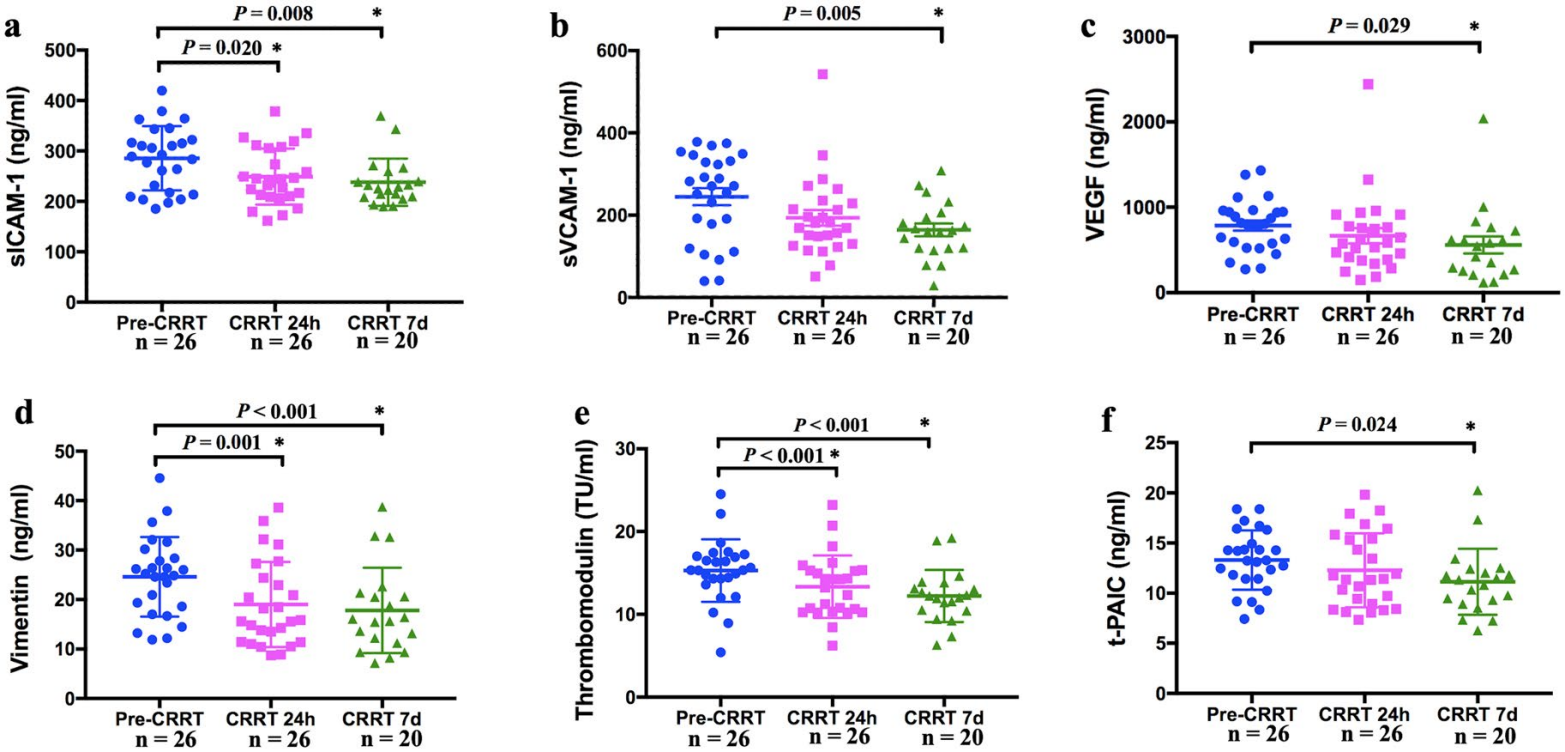
Comparison of serum endothelial cell injury indicators before and after CRRT intervention at 24 h and 7 days. (a) sICAM-1; (b) sVCAM-1; (c) VEGF; (d) Vimentin; (e) TM; (f) t-PAIC.

In the CRRT group, the values of sICAM-1, sVCAM-1, VEGF, VIM, TM, and t-PAIC were higher in the pre-CRRT period than those 7 days after CRRT (*p* = 0.008, *p* = 0.005, *p* = 0.029, *p* < 0.001, *p* < 0.001, and *p* = 0.024, respectively) (Figure 3). The levels of sICAM-1 VIM and TM decreased after 24 h CRRT compared with pre-CRRT (*p* = 0.020, *p* = 0.001, and *p* < 0.001, respectively) (Figure 3). The linear mixed-effects model revealed a statistically significant main effect of time on sICAM-1, sVCAM-1, VEGF, VIM, TM and t-PAIC levels [Wald *χ*^2^(2) = 10.28, *p* = 0.006; Wald *χ*^2^(2) = 9.91, *p* = 0.007; Wald *χ*^2^(2) = 6.12, *p* = 0.047; Wald *χ*^2^(2) = 19.01, *p* < 0.001; Wald *χ*^2^(2) = 21.57, *p* < 0.001, and Wald *χ*^2^(2) = 7.09, *p* = 0.029; respectively] (Table 2).

**Table 2.** Comparison of endothelial injury indicators during PICU stay.

CRRT group (*n* = 26)	Pre-CRRT (*N* = 26)	CRRT after 24 h (*N* = 26)	CRRT after 7 days (*N* = 20)	Wald *χ*²(2)	*p-*Value	Test
sICAM-1 (ng/ml)	290.85 (217.44, 322.19)	240.75 (210.26, 305.52)*	227.30 (209.29, 249.78)*	10.28	0.006	Wilcoxon
sVCAM-1 (ng/ml)	271.00 (178.90, 331.54)	175.40 (130.28, 228.64)	161.81 (119.85, 200.73)*	9.91	0.007	Wilcoxon
VEGF (ng/ml)	801.27 (577.45, 947.35)	581.54 (385.19, 776.09)	545.58 (255.34, 727.03)*	6.12	0.047	Wilcoxon
Vimentin (ng/ml)	25.04 (18.60, 28.34)	15.73 (11.44, 24.40)*	15.87 (11.73, 20.99)*	19.01	<0.001	Wilcoxon
TM (TU/ml)	15.28 ± 3.78	13.35 ± 3.76*	12.21 ± 3.16*	21.57	<0.001	Paired *t*-test
t-PAIC (ng/ml)	13.31 ± 2.96	12.28 ± 3.69	11.13 ± 3.29*	7.09	0.029	Paired *t*-test
**Non-CRRT group (*n* = 35)**	**Identified as sepsis (*N* = 35)**	**24 h after diagnosed with sepsis (*N* = 35)**	**7 days after diagnosed with sepsis (*N* = 35)**	**Wald *χ*²(2)**	***p-*Value**	
sICAM-1 (ng/ml)	326.34 (221.23, 433.55)	322.27 (230.11, 461.21)	262.82 (180.77, 376.88)	3.51	0.173	Wilcoxon
sVCAM-1 (ng/ml)	306.93 (216.27, 374.23)	296.45 (232.28, 421.77)	321.25 (229.22, 355.55)	2.05	0.359	Wilcoxon
VEGF (ng/ml)	634.21 (432.13, 872.10)	589.24 (341.90, 834.01)	523.45 (321.39, 790.25)	1.91	0.384	Wilcoxon
Vimentin (ng/ml)	22.57 (18.23, 25.62)	21.34 (12.11, 32.94)	18.34 (10.38, 30.24)	0.41	0.813	Wilcoxon
TM (TU/ml)	13.66 ± 0.62	12.17 ± 0.50*	11.99 ± 0.44*	8.97	0.011	Paired *t*-test
t-PAIC (ng/ml)	12.27 ± 0.50	10.73 ± 0.42*	11.12 ± 0.47	8.80	0.012	Paired *t*-test

sICAM-1: serum intercellular adhesion molecule-1; sVCAM-1: serum vascular cell adhesion molecule-1; t-PAIC: tissue plasminogen activator inhibitor-1 complex; TM: thrombomodulin; VEGF: vascular endothelial growth factor.

Values are expressed as the mean ± standard deviation or median (IQR).

*p < 0.05 indicates the significant difference compared with Pre-CRRT group.

In the non-CRRT group, the mixed-model analysis indicated a significant effect of time on TM and t-PAIC [Wald *χ*^2^(2) = 8.97, *p* = 0.011 and Wald *χ*^2^(2) = 8.80, *p* = 0.012, respectively]. However, the serum levels of sICAM-1, sVCAM-1, VEGF and VIM were not significantly different among the three time points (on PICU admission and confirmation of the presence of sepsis *vs.* 24 h after diagnosed with sepsis *vs.* 7 days after diagnosis with sepsis) [Wald *χ*^2^(2) = 3.51, *p* = 0.173; Wald *χ*^2^(2) = 2.05, *p* = 0.359; Wald *χ*^2^(2) = 1.91, *p* = 0.384; and Wald *χ*^2^(2) = 0.41, *p* = 0.813; respectively] (Table 2) (Supplementary Figure 1).

### Laboratory parameters in the CRRT group

The changes in the biochemical indices were shown in Table 3. The serum value of CRP, PCT, sCr and Lac were reduced gradually 7 days after CRRT (*p* < 0.001, *p* = 0.002, *p* = 0.008, and *p* = 0.001, respectively), the level of ALB was significantly increased at 7 days after CRRT (33.90 ± 5.67 *vs.* 39.71 ± 5.88 *vs.* 41.94 ± 5.22 g/L, *p* < 0.001) (Table 3). Moreover, serum IL-6 and IL-10 levels decreased 7 days after CRRT (*p* < 0.001 and *p* = 0.001, respectively). However, the serum levels of TNF-α, IL-1β, and IL-8 showed no significant decrease after CRRT (*p* = 0.959, *p* = 0.719, and *p* = 0.525, respectively) (Supplementary Table 1).

### The association of endothelial cell indicators with laboratory parameters at sepsis in the CRRT group

**Figure 4.**
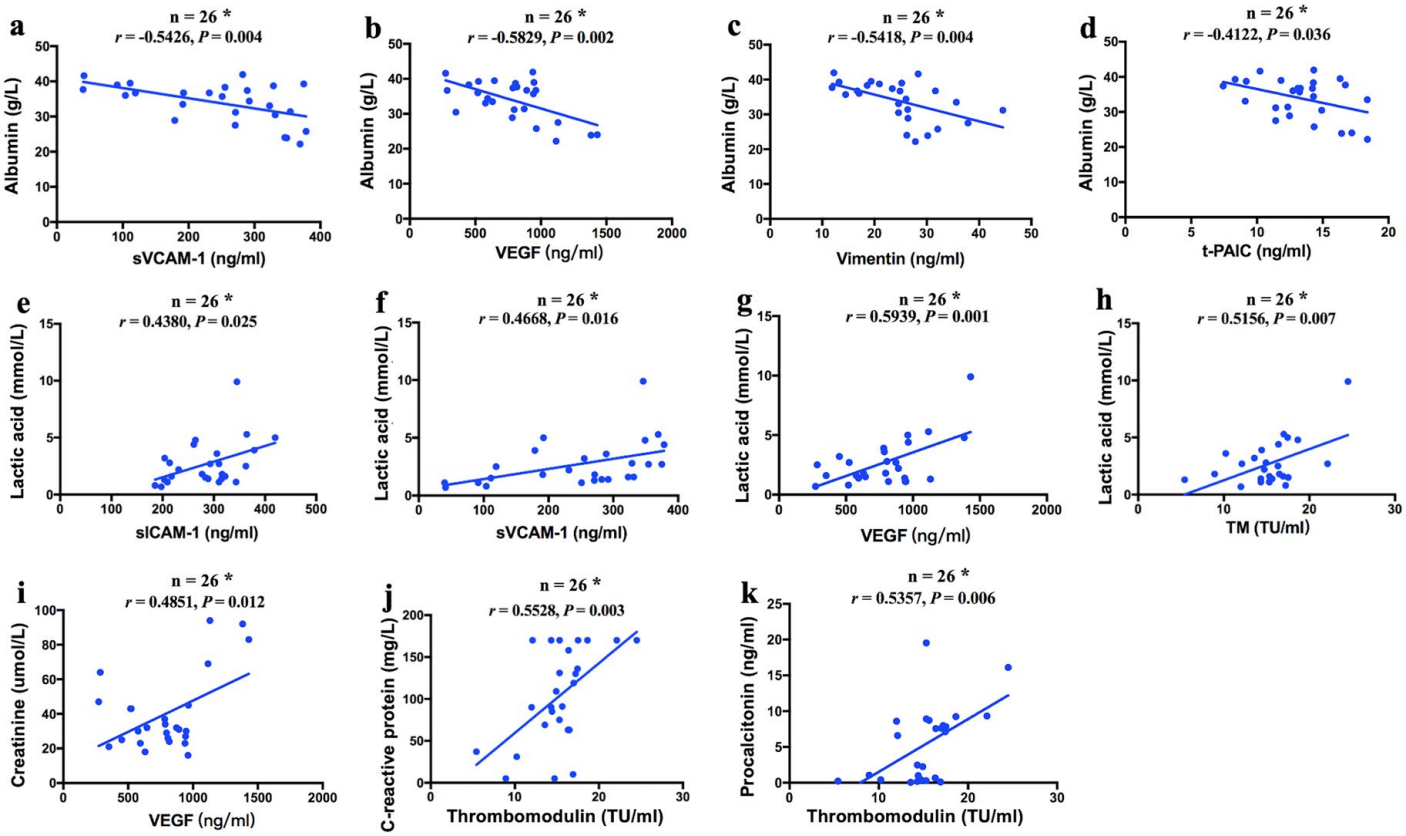
Correlations of serum endothelial cell injury indicators with laboratory parameters in the CRRT group at sepsis diagnosis. (a) ALB with sVCAM-1; (b) ALB with VEGF; (c) ALB with Vimentin; (d) ALB with t-PAIC; (e) Lac with sICAM-1; (f) Lac with sVCAM-1; (g) Lac with VEGF; (h) Lac with TM; (i) sCr with VEGF; (j) CRP with TM; (k) PCT with TM.

We then performed a correlation analysis to explore the relationship between EC indicators and indicators of organ function in the CRRT group at sepsis diagnosis. Based on the results of the correlation analysis, ALB levels were moderately negatively correlated with the levels of sVCAM-1, VEGF, VIM, and t-PAIC (*r* = −0.543, *p* = 0.004; *r* = −0.583, *p* = 0.002; *r* = −0.542, *p* = 0.004; *r* = −0.412, *p* = 0.036; respectively) (Figure 4). Serum levels of sICAM-1, sVCAM-1, VEGF, and TM were moderately positively correlated with Lac levels, which are well-known indicators of shock (*r* = 0.438, *p* = 0.025; *r* = 0.467, *p* = 0.016; *r* = 0.594, *p* = 0.001; *r* = 0.516, *p* = 0.007; respectively) (Figure 4). Moreover, there was a moderate correlation between indicators of kidney function (sCr) and VEGF (*r* = 0.485, *p* = 0.012) (Figure 4). Serum CRP and PCT levels were moderately correlated with TM (*r* = 0.553, *p* = 0.003; *r* = 0.536, *p* = 0.006; respectively) (Figure 4).

**Figure 5.**
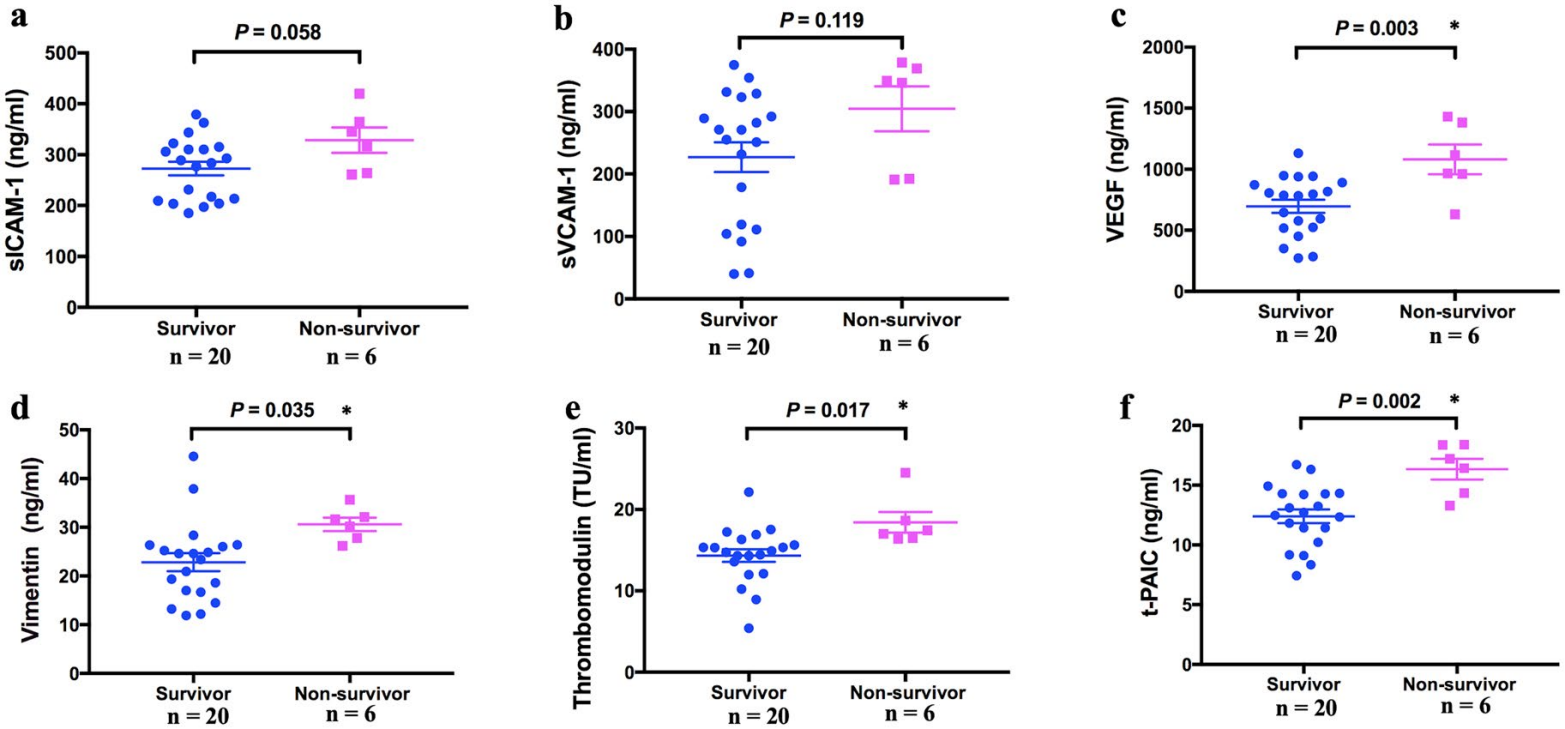
Comparison of serum endothelial cell indicators between survivors and non-survivors in the CRRT group at sepsis diagnosis. (a) ICAM-1; (b) VCAM-1; (c) VEGF; (d) Vimentin; (e) TM; (f) t-PAIC.

In the CRRT group, serum levels of sVCAM-1 and sICAM-1 displayed a decreasing trend in survivors compared with non-survivors at the time of sepsis diagnosis (*p* = 0.058, *p* = 0.119, respectively) (Figure 5). Among pediatric sepsis patients, serum VEGF, VIM, t-PAIC, and TM levels were significantly higher in non-survivors than in survivors (*p* = 0.003, *p* = 0.035, *p* = 0.002, and *p* = 0.017, respectively) (Figure 5).

## Discussion

The current study revealed that serum sICAM-1, sVCAM-1, VEGF, VIM, TM, and t-PAIC decreased after CRRT compared to those before CRRT. Moreover, the levels of EC injury indicators were related to the biomarkers of organ dysfunction in pediatric sepsis under CRRT, and serum indicators of EC injury in survivors were lower than those in non-survivors. To the best of our knowledge, this is the first report of indicators of EC injury in patients with sepsis who received CRRT.

Sepsis-induced EC dysfunction leads to coagulation dysfunction, impaired vascular permeability and tone, and impaired microcirculatory blood flow. EC injury ultimately contributed to organ dysfunction during sepsis [3,4]. Interdicting pathways that lead to sepsis-associated EC injury is a prospective avenue for improving sepsis-induced organ failure and mortality. One study showed extracorporeal hemoperfusion device (PMX-DHP) protects endothelial cells in adults with Gram-negative sepsis [17], and another study showed that serum from multiple organ dysfunction syndrome (MODS) patients induced endothelial dysfunction in HUVECs by increasing the secretion of specific factors, an effect conversely alleviated by CRRT [18]. In our study, serum levels of EC injury indicators in pediatric sepsis patients undergoing CRRT steadily decreased compared to those without CRRT. The reduction in these biomarkers may partly stem from overall clinical improvement or decreased inflammation, and partly from the clearance effect of hemodiafiltration (Graphical Abstract). Our further analysis found that the serum levels of sICAM-1, sVCAM-1, VEGF, VIM, TM, and t-PAIC were significantly different among the three groups (pre-CRRT *vs.* CRRT after 24 h *vs.* CRRT after 7 days, all *p* < 0.05). These results indicate that CRRT is effective for pediatric sepsis, partially contributing to the improvement of hemodynamic disorders and EC injury.

VEGF plays a crucial role in microvascular proliferation as an angiogenic cytokine factor that maintains vascular networks in tissues, including the kidney [19]. In particular, glomerular membrane endothelial cell fenestrations rely on VEGF-induced NO production, which contributes to preservation of the normal kidney barrier and filtration function [20]. In addition, we found a positive correlation between serum VEGF and sCr levels. Higher VEGF levels were correlated with elevated sCr, suggesting an association with kidney dysfunction rather than causation. Excessive proinflammatory cytokines produced during sepsis impair the vascular endothelial barrier [21]. The integrity of the endothelium is a sine qua non for the maintenance of tissue-fluid balance. Increased capillary permeability leads to increased albumin loss. Low serum albumin levels are commonly associated with organ dysfunction and poor outcomes [22]. Serum ALB levels were negatively correlated with sVCAM-1, VEGF, VIM, and t-PAIC levels (All *p* < 0.05). The ALB value gradually increased after CRRT (pre-CRRT *vs.* CRRT 24 h *vs.* CRRT 7 days) (*p* < 0.001), while sVCAM-1, VEGF, VIM, and t-PAIC levels significantly decreased after CRRT. Therefore, CRRT may improve sepsis-associated organ dysfunction, which is partially associated with improved EC dysfunction.

Growing evidence proved that endothelial biomarkers were closely relevant to the severity of illness in sepsis [23–25]. Elevated levels of endothelial biomarkers are associated with increased mortality [26]. Survivors had lower VEGF levels than did the non-survivors both in pediatric and adult patients [25–27]. High serum levels of vascular cell adhesion molecule-1 (VCAM-1) and ICAM-1 are associated with sepsis-induced organ dysfunction and in-hospital mortality in adult patients [28]. Our previous prospective observational study found that a high serum VIM level could predict a high risk of in-hospital mortality in children with sepsis [29]. Moreover, in pediatric sepsis patients receiving CRRT in our study, the serum levels of EC injury indicators (VEGF, VIM, TM, and t-PAIC) were significantly higher in non-survivors than in survivors. CRRT downregulates EC injury biomarkers to mitigate organ disorders. In randomized controlled trials, serum levels of endothelial biomarkers can be used as outcome endpoints to rapidly reflect the interventional effect of CRRT on EC function, thereby shortening the trial duration and reducing the required sample size.

The cutoff point of hemofiltration membranes in CRRT is ∼35 kDa [30,31]. However, sICAM-1, sVCAM-1, and TM are the most common biomarkers of endothelial injury and are often thought of as cell-surface adhesion molecules with molecular weights (MW) of 82 kDa, 90 kDa, and 75 kDa, respectively (Supplementary Table 15) [32–34]. The clearance capacity of the CRRT membrane for biomarkers can be quantitatively evaluated using the sieving coefficient (S) and clearance rate (K), which are recognized as reliable indices for assessing membrane permeability and actual removal efficiency. Specifically, a 35 kDa CRRT membrane exhibits almost no permeability to such large molecules, as reflected by an extremely low sieving coefficient (typically *S* ≤ 0.05) and a corresponding clearance rate close to zero. Therefore, sICAM-1, sVCAM-1, and TM could not be removed by all forms of CRRT. The decrease in the levels of endothelial injury indicators under CRRT treatment is potentially associated with regulation of endothelial signaling pathways. In our study, we found that VIM could be the first responder to CRRT, which was previously reported to be correlated with mortality in pediatric sepsis [29]. VIM, a novel indicator of EC injury, is predominantly present in the blood of patients with sepsis (MW 57 kDa) [35]. VIM production in HUVECs might be modulated through the TGF-β/Smad signaling pathway [36–38]. TGF-β is a multifunctional growth factor and has the ability to induce cell proliferation, differentiation, and programmed cell death [39–41]. Given its low molecular weight (≈12.5 kDa), TGF-β may be partially cleared during CRRT; this could contribute to decreased VIM expression [42,43]. This may be the reason why patients with sepsis showed decreased VIM levels after CRRT.

Sepsis-induced glycocalyx damage and Ang/Tie2 signaling imbalance lead to heightened endothelial permeability and compromised endothelial barrier function [44,45]. VEGF impairs the integrity of the endothelial glycocalyx through two distinct mechanisms: enzymatic degradation of glycosaminoglycans within the glycocalyx structure and activation of the Rho/ROCK signaling pathway, ultimately contributing to vascular hyperpermeability [46,47]. TM, a marker of endothelial injury, in turn, further exacerbates Ang/Tie2 signaling pathway dysfunction upon its shedding [48]. Consequently, this study’s finding that CRRT lowered circulating EC injury biomarkers (e.g., VEG and TM) and mitigated endothelial injury points to these biomarkers’ potential involvement in the repair and degradation processes of the endothelial glycocalyx, as well as their role in regulating the Ang/Tie2 signaling pathway.

We can also set the levels of endothelial biomarkers as the indicator for initiating CRRT in sepsis, as endothelial biomarkers, serving as early markers reflecting EC injury and organ dysfunction in sepsis, are superior to the timing of CRRT initiation based on traditional organ failure indicators (e.g., hyperkalemia, acidosis, renal injury, etc.). Meanwhile, during CRRT, dynamic monitoring of EC injury biomarkers reflects real-time endothelial injury or recovery in sepsis, aiding in evaluating sepsis severity and optimizing CRRT dose or mode. We can further optimize endothelial function, construct artificial biological membranes replicating endothelial glycocalyx structural features. The levels of relevant markers can serve as key indicators for evaluating membrane functional performance. Additionally, artificial intelligence (AI) is revolutionizing the management of sepsis and other complex conditions in critically ill patients, addressing key challenges in critical care medicine [49,50]. AI can stratify AKI patients into sub-phenotypes with distinct clinical features and outcomes, enabling tailored interventions [51]. ML models enable early prediction of AKI, up to 48 h earlier than traditional serum creatinine monitoring [51]. Therefore, we may prospectively build predictive models using AI algorithms, define biomarker thresholds, and support the CRRT clinical decision-making workflow in the future.

The limitations of this study should be discussed. First, this was a small, single-center study. Secondly, data on the CRRT dose-response relationship was absent. We only performed CRRT with a dose delivered of 35 mL/kg/h without data on different ultrafiltration dosages of CRRT. Thirdly, an important limitation was the potential for confounding by indication. Additionally, we did not control for vasoactive or anti-inflammatory therapies. Lastly, there was a lack of data on long-term outcomes. A large-scale prospective multicenter study should be conducted to further confirm this result in the future.

## Conclusion

Patients with higher serum VEGF, VIM, TM, and t-PAIC levels were associated with worse outcomes for pediatric sepsis. CRRT downregulated the serum levels of EC injury biomarkers.

## Supplementary Material

Supplemental Material

Supplementary Figure 1.png

## Data Availability

The anonymized raw dataset, cleaned dataset, and statistical analysis code of this study have been archived in the open science platform [Figshare], with the unique identifier DOI: 10.6084/m9.figshare.30812312. The resources are publicly retrievable *via* the platform to ensure the reproducibility of the study data and methods.
