# Supplementary material for: Continuous renal replacement therapy attenuates endothelial injury biomarkers in pediatric sepsis: a prospective cohort study
Source: Ren Fail. 2026 Mar 31;48(1):2639791. doi: 10.1080/0886022X.2026.2639791 (PMC13040570; doi:10.1080/0886022X.2026.2639791)
Supplement: Supplemental Material [file IRNF_A_2639791_SM6357.docx]

**Supplementary Table 1. Comparison of the percent of immune cells and cytokines in CRRT group**

|  | **Pre-CRRT**  **(N = 26)** | **CRRT after 24h**  **(N = 26)** | **CRRT after 7d**  **(N = 20)** | ***p-*value** |
| --- | --- | --- | --- | --- |
| NK (%) | 5.59 (3.10, 8.80) | 2.38 (1.52, 3.81) * | 3.08 (1.51, 4.87) * | 0.004 |
| CD19 (%) | 32.61 (26.61, 49.53) | 42.72 (33.43, 45.90) | 22.04 (13.96, 36.89) | 0.145 |
| CD4+CD8+ (%) | 1.81 (1.13, 2.65) | 1.43 (0.98, 1.84) | 1.80 (1.13, 2.23) | 0.301 |
| IL-1β (pg/ml) | 7.15 (0.10, 38.71) | 0.10 (0.10, 18.18) | 0.10 (0.10, 9.84) | 0.719 |
| IL-6 (pg/ml) | 598.00 (29.84, 4677.00) | 15.57 (5.04, 1357.60) | 8.74 (0.10, 22.43) *^#^ | ＜0.001 |
| IL-8 (pg/ml) | 48.15 (0.10, 94.60) | 49.91 (9.15, 116.62) | 49.26 (26.55, 108.76) | 0.525 |
| IL-10 (pg/ml) | 11.37 (5.22, 519.07) | 6.49 (0.10, 104.89) | 0.10 (0.10, 3.21) *^#^ | 0.001 |
| TNF-α (pg/ml) | 0.10 (0.10, 5.89) | 0.10 (0.10, 5.50) | 0.10 (0.10, 6.51) | 0.959 |

NK: natural killer cells; IL-1β: interleukin-1β; IL-6: interleukin-6; IL-8: interleukin-8; IL-10: interleukin-10; TNF-α: tumor necrosis factor-α; values are expressed as the median (IQR).

^*^*P* < 0.05 indicates the significant difference compared with Pre-CRRT group. ^#^*P* < 0.05 indicates the significant difference compared with CRRT after 24h group.

**Supplementary Table 2. Percentage reduction in EC injury indicators with 95% confidence intervals (CIs) and Cohen’s d**

| **CRRT group (N = 26)** | **Percentage reduction at 24h post-CRRT (%)** | **95%CI** | **Cohen’s d** | **Percentage reduction at 7d post-CRRT (%)** | **95%*CI*** | **Cohen’s d** |
| --- | --- | --- | --- | --- | --- | --- |
| sICAM-1 (ng/ml) | 12.08 | -64.04 to -8.68 | -0.53 | 38.49 | -74.13 to -14.13 | -0.48 |
| sVCAM-1 (ng/ml) | 20.23 | -98.21 to -4.09 | -0.37 | 49.97 | -129.83 to -28.04 | -0.52 |
| VEGF (ng/ml) | 14.05 | -278.64 to 41.50 | -0.24 | 49.55 | -400.50 to -43.79 | -0.48 |
| Vimentin (ng/ml) | 22.13 | -8.45 to -2.79 | -0.68 | 47.30 | -8.68 to -2.47 | -0.77 |
| TM (TU/ml) | 12.41 | -2.85 to -1.02 | -0.99 | 40.97 | -2.94 to -0.93 | -0.63 |
| t-PAIC (ng/ml) | 7.75 | -2.07 to 0.01 | -0.47 | 38.91 | -2.61 to -0.32 | -0.45 |
| **Non-CRRT group (N = 35)** | **Percentage reduction at 24h post-CRRT (%)** | **95%CI** | **Cohen’s d** | **Percentage reduction at 7d post-CRRT (%)** | **95%*CI*** | **Cohen’s d** |
| sICAM-1 (ng/ml) | -6.88 | -51.22 to 97.47 | 0.09 | 13.87 | -120.99 to 27.67 | -0.22 |
| sVCAM-1 (ng/ml) | -11.09 | -27.06 to 93.74 | 0.17 | 2.78 | -68.74 to 52.06 | -0.05 |
| VEGF (ng/ml) | 7.61 | -170.26 to 71.26 | -0.16 | 13.04 | -205.60 to 35.93 | -0.20 |
| Vimentin (ng/ml) | 0.68 | -4.84 to 4.54 | -0.01 | 6.27 | -6.09 to 3.27 | -0.10 |
| TM (TU/ml) | 10.96 | -2.70 to -0.29 | -0.48 | 12.28 | -2.88 to -0.47 | -0.39 |
| t-PAIC (ng/ml) | 12.57 | -2.60 to -0.48 | -0.57 | 9.37 | -2.21 to -0.09 | -0.33 |

**Supplementary Table 3. Sensitivity analyses of vimentin levels in CRRT group**

| **Vimentin** | **Model_full** | **Model_noout** | **Model_winsor** |
| --- | --- | --- | --- |
| CRRT after 24h | -5.6195305*** | -5.6195305*** | -5.6195304*** |
| CRRT after 7d | -5.5740599*** | -5.5740599*** | -0.63692179 |
| Intercept (cons) | 24.62366*** | 24.62366*** | 24.62366*** |
| lnl1_1_1 | 1.8753707*** | 1.8753707*** | 2.0177766*** |
| lnsig_e | 1.6269485*** | 1.6269485*** | 1.9394744*** |
| Sample size (N) | 72 | 72 | 78 |
| Log-likelihood (ll) | -241.47189 | -241.47189 | -281.53403 |

Vimentin baseline level remained highly stable. The short-term effect of CRRT (24 hours) demonstrated good robustness in influencing vimentin levels, while the long-term effect (7 days) may be considerably influenced by outliers.

^*^*P* < 0.05. ^**^*P* < 0.01. ^***^*P* < 0.001.

**Supplementary Table 4. Sensitivity analyses of sICAM-1 levels in CRRT group**

| **sICAM-1** | **Model_full** | **Model_noout** | **Model_winsor** |
| --- | --- | --- | --- |
| CRRT after 24h | -36.362862** | -36.362862** | -36.362863* |
| CRRT after 7d | -44.11906** | -44.11906** | -5.5069879 |
| Intercept (cons) | 285.52519*** | 285.52519*** | 285.52519*** |
| lnl1_1_1 | 3.1975568*** | 3.1975568*** | 3.631622*** |
| lnsig_e | 3.9086095*** | 3.9086095*** | 4.0612179*** |
| Sample size (N) | 72 | 72 | 78 |
| Log-likelihood (ll) | -390.20872 | -390.20872 | -438.11227 |

The baseline level of sICAM-1 was highly stable. The short-term effect of CRRT (24 hours) demonstrated good robustness in influencing sICAM-1 levels, whereas the long-term effect of CRRT (7 days) on sICAM-1 levels was considerably influenced by outliers.

^*^*P* < 0.05. ^**^*P* < 0.01. ^***^*P* < 0.001.

**Supplementary Table 5. Sensitivity analyses of sVCAM-1 levels in CRRT group**

| **sVCAM-1** | **Model_full** | **Model_noout** | **Model_winsor** |
| --- | --- | --- | --- |
| CRRT after 24h | -51.148316* | -64.695273** | -51.14832 |
| CRRT after 7d | -78.925778** | -78.797353*** | 6.5233218 |
| Intercept (cons) | 244.92252*** | 244.92252*** | 244.92252*** |
| lnl1_1_1 | 3.5805426*** | 3.3680079*** | 3.910424*** |
| lnsig_e | 4.4394029*** | 4.3468688*** | 4.7663293*** |
| Sample size (N) | 72 | 71 | 78 |
| Log-likelihood (ll) | -427.02831 | -413.59767 | -488.0777 |

The baseline level of sVCAM-1 was highly stable. The short-term effect of CRRT on VCAM levels at 24 hours was relatively robust, while the effect at 7 days post-CRRT was interfered by outliers.

^*^*P* < 0.05. ^**^*P* < 0.01. ^***^*P* < 0.001.

**Supplementary Table 6. Sensitivity analyses of VEGF levels in CRRT group**

| **VEGF** | **Model_full** | **Model_noout** | **Model_winsor** |
| --- | --- | --- | --- |
| CRRT after 24h | -118.57327 | -187.47917** | -118.57327 |
| CRRT after 7d | -222.13369* | -295.68323*** | 280.75253 |
| Intercept (cons) | 785.173*** | 785.173*** | 785.173*** |
| lnl1_1_1 | 5.56886*** | 5.0686165*** | 5.5698294*** |
| lnsig_e | 5.6628689*** | 5.4274985*** | 6.3120861*** |
| Sample size (N) | 71 | 69 | 78 |
| Log-likelihood (ll) | -518.09141 | -483.10036 | -609.76298 |

The baseline level of VEGF was highly stable. The short-term effect of CRRT on VEGF changes at 24 hours had certain stability, while the effect on VEGF at 7 days after CRRT was extremely strongly interfered by outliers.

^*^*P* < 0.05. ^**^*P* < 0.01. ^***^*P* < 0.001.

**Supplementary Table 7. Sensitivity analyses of TM levels in CRRT group**

| **TM** | **Model_full** | **Model_noout** | **Model_winsor** |
| --- | --- | --- | --- |
| CRRT after 24h | -1.9303846*** | -1.9303846*** | -1.9303848** |
| CRRT after 7d | -1.9338108*** | -1.9338108*** | -0.23076921 |
| Intercept (cons) | 15.276154*** | 15.276154*** | 15.276154*** |
| lnl1_1_1 | 1.2041082*** | 1.2041082*** | 1.3450339*** |
| lnsig_e | 0.49827439*** | 0.49827439*** | 0.87625378*** |
| Sample size (N) | 72 | 72 | 78 |
| Log-likelihood (ll) | -170.5827 | -170.5827 | -207.09014 |

The baseline level of TM was highly stable. The short-term effect of CRRT (24 hours) on TM was highly robust, while the long-term effect on TM at 7 days post-CRRT was significantly affected by outliers.

^*^*P* < 0.05. ^**^*P* < 0.01. ^***^*P* < 0.001.

**Supplementary Table 8. Sensitivity analyses of t-PAIC levels in CRRT group**

| **t-PAIC** | **Model_full** | **Model_noout** | **Model_winsor** |
| --- | --- | --- | --- |
| CRRT after 24h | -1.0284615* | -1.0284615* | -1.0284613 |
| CRRT after 7d | -1.4659051* | -1.4659051* | -0.06830744 |
| Intercept (cons) | 13.306154*** | 13.306154*** | 13.306154*** |
| lnl1_1_1 | 1.0138682*** | 1.0138682*** | 1.1225885*** |
| lnsig_e | 0.62820167*** | 0.62820167*** | 0.83507814*** |
| Sample size (N) | 72 | 72 | 78 |
| Log-likelihood (ll) | -172.54444 | -172.54444 | -199.80529 |

The baseline level of t-PAIC was highly stable. The robustness of the short-term effect of CRRT (24 hours) on t-PAIC was moderate, while the long-term effect of CRRT (7 days) on t-PAIC was extremely strongly interfered by outliers.

^*^*P* < 0.05. ^**^*P* < 0.01. ^***^*P* < 0.001.

**Supplementary Table 9. Sensitivity analyses of vimentin levels in Non-CRRT group**

| **Vimentin** | **Model_full** | **Model_noout** | **Model_winsor** |
| --- | --- | --- | --- |
| 24 hours after diagnosed with sepsis | -0.15130429 | -0.15130429 | -0.13736138 |
| 7 days after diagnosed with sepsis | -1.4014786 | -1.4014786 | -1.4091929 |
| Intercept (cons) | 22.334879*** | 22.334879*** | 22.334879*** |
| lnl1_1_1 | 1.2283751* | 1.2283751* | 1.2303356* |
| lnsig_e | 2.2887797*** | 2.2887797*** | 2.2870126*** |
| Sample size (N) | 105 | 105 | 105 |
| Log-likelihood (ll) | -394.68887 | -394.68887 | -394.53794 |

The baseline level of Vimentin was highly stable. Non-statistically significant negative effects on Vimentin were observed at 24 hours and 7 days post-treatment. Among them, the short-term effect (24 hours post-treatment) showed slight numerical fluctuations but a stable direction, while the long-term effect (7 days post-treatment) was not affected by outliers.

^*^*P* < 0.05. ^**^*P* < 0.01. ^***^*P* < 0.001.

**Supplementary Table 10. Sensitivity analyses of sICAM-1 levels in Non-CRRT group**

| **sICAM-1** | **Model_full** | **Model_noout** | **Model_winsor** |
| --- | --- | --- | --- |
| 24 hours after diagnosed with sepsis | 23.127062 | 23.127062 | 22.691917 |
| 7 days after diagnosed with sepsis | -46.646366 | -46.646366 | -46.340387 |
| Intercept (cons) | 336.21408*** | 336.21408*** | 336.21408*** |
| lnl1_1_1 | -14.81126*** | -14.81126*** | -13.766553** |
| lnsig_e | 5.0523731*** | 5.0523731*** | 5.0490059*** |
| Sample size (N) | 105 | 105 | 105 |
| Log-likelihood (ll) | -679.48772 | -679.48772 | -679.13417 |

The baseline level of sICAM-1 was highly stable. The short-term effect (24 hours post-treatment) exerted a stable directional impact on sICAM-1 with no statistical significance and low susceptibility to outliers; the long-term effect (7 days post-treatment) showed stable direction and magnitude of impact on sICAM-1 with minimal outlier interference.

^*^*P* < 0.05. ^**^*P* < 0.01. ^***^*P* < 0.001.

**Supplementary Table 11. Sensitivity analyses of sVCAM-1 levels in Non-CRRT group**

| **sVCAM-1** | **Model_full** | **Model_noout** | **Model_winsor** |
| --- | --- | --- | --- |
| 24 hours after diagnosed with sepsis | 33.338163 | 21.622448 | 30.235296 |
| 7 days after diagnosed with sepsis | -8.342123 | -8.342123 | -8.3421302 |
| Intercept (cons) | 300.55755*** | 300.55755*** | 300.55756*** |
| lnl1_1_1 | -17.769851*** | -20.602595*** | -17.698423*** |
| lnsig_e | 4.8447103*** | 4.7988407*** | 4.8211579*** |
| Sample size (N) | 105 | 104 | 105 |
| Log-likelihood (ll) | -657.68313 | -646.64904 | -655.21013 |

The baseline level of sVCAM-1 was highly stable. The short-term effect (24 h post-treatment) on sVCAM-1 was significantly affected by individual outliers with insufficient robustness; the long-term effect (7 d post-treatment) was minimally interfered by outliers with strong stability.

^*^*P* < 0.05. ^**^*P* < 0.01. ^***^*P* < 0.001.

**Supplementary Table 12. Sensitivity analyses of VEGF levels in Non-CRRT group**

| **VEGF** | **Model_full** | **Model_noout** | **Model_winsor** |
| --- | --- | --- | --- |
| 24 hours after diagnosed with sepsis | -49.496286 | -49.496286 | -49.823141 |
| 7 days after diagnosed with sepsis | -84.834857 | -84.834857 | -85.07742 |
| Intercept (cons) | 650.554*** | 650.554*** | 650.79657*** |
| lnl1_1_1 | 4.8160498*** | 4.8160498*** | 4.8145499*** |
| lnsig_e | 5.5374911*** | 5.5374911*** | 5.5368175*** |
| Sample size (N) | 105 | 105 | 105 |
| Log-likelihood (ll) | -739.80082 | -739.80082 | -739.7181 |

The baseline level of VEGF was highly stable. Both the 24-hour and 7-day post-treatment effects on VEGF levels exhibited non-statistically significant negative effects, with the magnitude and direction of both effects unaffected by outliers and showing strong robustness.

^*^*P* < 0.05. ^**^*P* < 0.01. ^***^*P* < 0.001.

**Supplementary Table 13. Sensitivity analyses of TM levels in Non-CRRT group**

| **TM** | **Model_full** | **Model_noout** | **Model_winsor** |
| --- | --- | --- | --- |
| 24 hours after diagnosed with sepsis | -1.4979714* | -1.4979714* | -1.456257* |
| 7 days after diagnosed with sepsis | -1.6781143** | -1.6781143** | -1.661257** |
| Intercept (cons) | 13.663143*** | 13.663143*** | 13.646286*** |
| lnl1_1_1 | 0.54456012* | 0.54456012* | 0.5317623* |
| lnsig_e | 0.93085473*** | 0.93085473*** | 0.92250779*** |
| Sample size (N) | 105 | 105 | 105 |
| Log-likelihood (ll) | -261.94256 | -261.94256 | -260.97582 |

The baseline level of TM was highly stable. Both the 24-hour and 7-day post-treatment effects on TM levels showed significant negative effects (with a more pronounced effect at 7 days post-treatment), and neither effect was interfered by outliers, demonstrating extremely strong robustness.

^*^*P* < 0.05. ^**^*P* < 0.01. ^***^*P* < 0.001.

**Supplementary Table 14. Sensitivity analyses of TM levels in Non-CRRT group**

| **t-PAIC** | **Model_full** | **Model_noout** | **Model_winsor** |
| --- | --- | --- | --- |
| 24 hours after diagnosed with sepsis | -1.5426486** | -1.5426486** | -1.4986485** |
| 7 days after diagnosed with sepsis | -1.1497429* | -1.1497429* | -1.1197427* |
| Intercept (cons) | 12.268286*** | 12.268286*** | 12.238286*** |
| lnl1_1_1 | 0.42446672* | 0.42446672* | 0.41798339* |
| lnsig_e | 0.80157737*** | 0.80157737*** | 0.78813562*** |
| Sample size (N) | 105 | 105 | 105 |
| Log-likelihood (ll) | -248.55584 | -248.55584 | -247.28741 |

The baseline level of t-PAIC was highly stable. The significant negative effect of 24-hour post-treatment on t-PAIC was robust (unaffected by outliers), while the negative effect remained significant at 7 days post-treatment but was susceptible to outliers with weak robustness.

^*^*P* < 0.05. ^**^*P* < 0.01. ^***^*P* < 0.001.

**Supplementary Table 15. The biology information of EC injury indicators**

| **Endothelial cell injury biomarker** | **Function** | **MW (KDa)** | **Mechanistic relevance** | **Direction of change** | **Clinical significance** |
| --- | --- | --- | --- | --- | --- |
| sICAM-1 | Mediates firm adhesion of leukocytes to the endothelium | 82 | Upon endothelial activation, ICAM-1is shed from the cell surface and released into the bloodstream. Disrupt the barrier function of endothelial cells, resulting in increased vascular permeability | Increased | Marker of Endothelial Activation. Levels correlate with disease severity and organ failure scores. Persistently high levels predict poor outcomes |
| sVCAM-1 | Mediates the adhesion of lymphocytes and monocytes to the endothelium | 90 | Similar to sICAM-1, its expression increases upon endothelial activation and is shed. Its expression is induced by pro-inflammatory cytokines | Increased | Marker of Endothelial Activation in chronic/immune inflammation. Also elevated in sepsis, has potential value in differentiating types of inflammation |
| VEGF | Promotes angiogenesis and endothelial cell survival | 45 | Upregulated in the early stages of sepsis. A sharp increase in VEGF mediates vascular leakage and tissue edema | Increased | The "Master Switch" for Vascular Leak. Early sharp rise directly contributes to shock and edema |
| Vimentin | A type III intermediate filament protein that maintains cellular shape and integrity | 57 | Released into the bloodstream upon cell necrosis or severe injury. Interaction with inflammatory signaling pathways affect the release of inflammatory mediators | Increased | Marker of Endothelial Injury/Death. Elevated levels indicate more severe structural damage. A powerful predictor of mortality |
| TM | A membrane-bound cofactor that binds thrombin to activate protein C, exerting a potent anticoagulant effect | 75 | Upon endothelial injury, TM is shed from the endothelial surface, leading to elevated soluble TM levels which promote the development of DIC | Increased | Marker of Endothelial Injury & Pro-coagulant State. A key indicator for diagnosing DIC and predicting outcomes. High levels strongly correlate with multi-organ failure and death risk |
| t-PAIC | Reflects the state of the fibrinolytic system, t-PA initiates fibrinolysis, PAI-1 inhibits it | 70 | Upon endothelial injury, increased release of PAI-1 binds to t-PA, forming a stable t-PAIC complex, leading to the inhibition of fibrinolysis | Increased | Marker of Fibrinolytic Shutdown. A characteristic feature of sepsis-induced DIC with suppressed fibrinolysis. Levels are highly correlated with sepsis severity and mortality |
