# Supplementary figures and images for: Continuous renal replacement therapy attenuates endothelial injury biomarkers in pediatric sepsis: a prospective cohort study
Source: Ren Fail. 2026 Mar 31;48(1):2639791. doi: 10.1080/0886022X.2026.2639791 (PMC13040570; doi:10.1080/0886022X.2026.2639791)

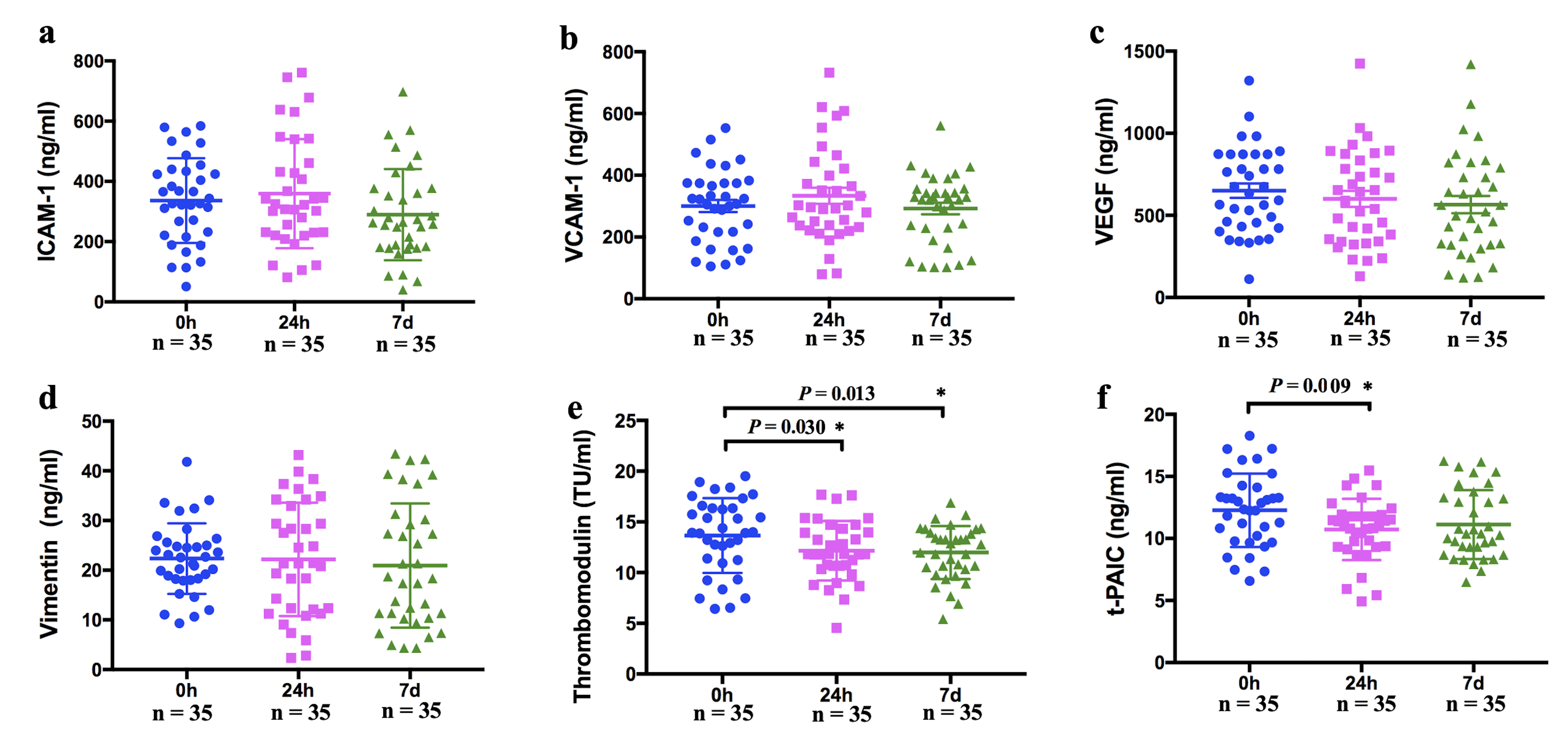

Supplement: Supplementary Figure 1.png [file IRNF_A_2639791_SM0233.png]
